# Digital Mental Health and Neurodevelopmental Services: Case-Based Realist Evaluation

**DOI:** 10.2196/29845

**Published:** 2021-09-17

**Authors:** Frank R Burbach, Katie M Stiles

**Affiliations:** 1 Healios Ltd London United Kingdom; 2 University of Exeter Exeter United Kingdom

**Keywords:** telehealth, young people, adolescents, online psychological therapy, online neurodevelopmental assessments, digital services, realist evaluation, multiple case study, CBT, autism

## Abstract

**Background:**

The rapid movement of mental health services on the internet following the onset of the COVID-19 pandemic has demonstrated the potential advantages of digital delivery and has highlighted the need to learn from prepandemic digital services.

**Objective:**

The aim of this study is to explore the different elements of interconnected digital mental health and neurodevelopmental services of a well-established provider to the UK National Health Service and how web-based delivery enables young people and their families to access high-quality assessments and interventions in a more timely, flexible, and person-centered manner than in-person delivery.

**Methods:**

A realist evaluation multiple case–study design was used, with 9 pediatric cases (aged 8-15 years) identified as representative of the services provided by Healios. Presenting concerns included autism and ADHD, anxiety and panic attacks, low self-esteem, anger and self-harm. The research literature was used to define the program theory and six context-mechanism-outcome (CMO) statements. The CMOs formed the basis for the initial data extraction, with novel elements added via an iterative process.

**Results:**

We identified 10 key elements of web-based services: flexible delivery and timely response, personalized care to the individual, comprehensive care enabled by multiple interconnected services, effective client engagement and productive therapeutic alliances, use of multiple communication tools, client satisfaction with the service, good clinical outcomes, ease of family involvement throughout sessions or from different locations, facilitation of multi-agency working and integration with National Health Services, and management of risk and safeguarding. These elements supported the six CMOs; there was clear evidence that young people and their families valued the responsiveness and flexibility of the web-based mental health service and, in particular, how quickly they were seen. There was also clear evidence of individual needs being met, good therapeutic alliances, and client satisfaction. Multiple communication tools appeared to maximize engagement and working digitally facilitated multi-agency communication and delivery of safe care. The abovementioned factors may be related to the finding of good clinical outcomes, but the methodology of this study does not allow any conclusions to be drawn regarding causality.

**Conclusions:**

This study demonstrates the effectiveness of interconnected digital mental health and neurodevelopmental services as well as how web-based delivery enables young people and their families to access assessments and interventions in a more timely, flexible, and person-centered manner than in-person delivery. The 10 key elements of web-based service delivery identified through the 9 case studies suggest the potential advantages of web-based work. These elements can inform future research and aid in the delivery of high-quality digital services.

## Introduction

### Background

The onset of the COVID-19 pandemic has forced services to be delivered on the web and given impetus to digital health care as a solution to access gaps by expanding and leveraging existing technologies [1,2]. In particular, although people with existing mental health problems have been resilient during the pandemic [3], many children and young people (CYP) have experienced low levels of anxiety and depression [4], exacerbating ongoing problems in accessing services [5]. Hence, a “digital revolution” [6] has been accelerated, with calls for the consolidation of gains [7] and the need to learn from prepandemic digital services [8]. A review of prepandemic literature concluded that “tele-mental health has potential to be an effective and acceptable form of service delivery” and called for future digital mental health implementation to use a combination of previous evidence and COVID-19 experiences [8]. Nicholas et al [9] reviewed children and adolescent mental health services (CAMHS) during the pandemic in Australia and noted that young people reported web-based services to have improved service quality, whereas the clinicians were significantly less positive.

The implications of web-based mental health services have previously been examined within the rubric of the ethics of digital delivery. In a narrative review of mostly North American literature, advantages were identified, including convenience, increased acceptance and adherence, cost efficiencies, enhancements in communication, and other therapy benefits [10]. Furthermore, factors contributing to the positive evaluation of web-based care by clients included meeting needs in a timely and effective manner [11], a more egalitarian therapeutic alliance [12], and an increased sense of being in control [9,13].

### This Study

This paper contributes to the literature on the effectiveness of digital service delivery by Healios, which has been providing a range of web-based CYP mental health and neurodevelopmental (ND) services to the UK National Health Service (NHS) since 2015. We purposely adopted a realist evaluation (RE) approach [14,15] to assess whether our service works in a particular context. In this case, we designed a digital service to maximize the advantages of providing web-based mental health services, and, in line with RE practice, we initially examined those case examples that involved different services and service combinations. The context is the provision of web-based youth mental health and ND services before the pandemic to inform development in the future.

In line with the RE methodology, we developed an initial program theory and context-mechanism-outcome (CMO) statements to enable data collection to focus on testing the different elements of the program theory. Our RE *mid-range theory* is that providing services digitally allows young people and their families to access mental health and ND services in a more timely, flexible, and person- and family- centered manner than in-person delivery. All the existing qualities of in-person service delivery, including the formation of a therapeutic alliance, can be delivered safely and effectively via the internet; clinical outcomes are equivalent to or better than in-person care, and additional efficiencies can be achieved. Digital delivery also improves outcome measurements and interagency communication.

Using themes primarily from recent reviews [9,10] and previous research suggesting that mental health service outcomes are associated with therapeutic alliance and client satisfaction [16,17], the authors and colleagues created CMO statements for Healios’ web-based services:

1. Timely provision of mental health services leads to better engagement and outcomes.
2. Personalized and flexible care leads to successfully building therapeutic alliance and client satisfaction.
3. Access to multiple interconnected services leads to more comprehensive care that meets the individual’s needs.
4. Digital mental health provides a more egalitarian experience than in-person mental health and leads to the empowerment of the client and better outcomes.
5. Using multiple digital communication tools (eg, videoconferencing, therapeutic information, interactive whiteboards, rating scales, and outcome measures) can enhance engagement and the therapeutic alliance.
6. Web-based communication facilitates safe care, networking, and access to therapeutic resources: particularly, information, support from peers and family, and liaison with schools and other agencies.

The goals of this study are (1) to analyze selected cases to explore the CMO statements and (2) to obtain insights into the delivery of high-quality web-based mental health care and ND services.

### Overview of Healios Services

Healios is a fully remote company using a bespoke electronic patient record system and remote delivery system, Panacea, which uses a cloud-based Ruby on Rails application hosted by Amazon Web Services. This specialized, secure platform (ISO [International Organization for Standardization] 27001 certified) has been developed over the past 8 years. It facilitates referral and client management, including a portal for the NHS to securely make referrals and monitor the progress of cases, the delivery of interventions and recording of sessions.

Accredited clinicians deliver all assessments and interventions through Panacea via split-screen videoconferencing, facilitating interaction with a clinician and interactive slides. Although clinicians adapt sessions to meet client needs, slide sets are provided to structure all clinical sessions. For example, a 10-session cognitive behavioral therapy (CBT) deck provides structure and ensures fidelity to the National Institute for Health and Care Excellence–endorsed (2017) CYP CBT manual, but additional sessions can be offered and some sessions can be omitted. The British Association for Behavioral and Cognitive Psychotherapies–accredited therapists can choose interactive CBT decks covering panic disorder, generalized anxiety, obsessive compulsive disorder, and depression. The other therapy package is goal-based intervention (GBI; maximum 6 sessions) delivered by CAMHS professionals or psychological well-being practitioners for mild-moderate presentations, especially where the young person is more practically oriented. In some cases, *getting help* or *getting more help* assessments (CAMHS tier 2 or 3 initial assessments) are offered to young people to ascertain whether CBT or GBI would be appropriate.

The Healios autism assessment service involves an extensive 5+ hours–long digital assessment process involving 2 clinical professionals conducting 3 videoconferencing clinical interviews with the young person and their parents based on 2 gold standard–validated assessment tools (Autism Diagnostic Interview-Revised and Autism Diagnostic Observation Schedule) [18] and clinical information obtained from schools, NHS, and social services, as appropriate. A video-conferencing multidisciplinary team, comprising a minimum of 3 clinicians, makes a diagnostic decision using the Diagnostic and Statistical Manual of Mental Disorders-5 criteria, followed by feedback to parents with advice on responding to their child’s needs and a report with guidance for teachers and other professionals. The attention-deficit/hyperactivity disorder (ADHD) assessment service is similar, and both may be preceded by an initial screening appointment. The postdiagnostic intervention (PDI) comprises up to 10 sessions of tailored content adapted to the needs of the young people and their families.

In addition to the abovementioned CYP services, Healios also offers adult ND services, including autism and ADHD assessments, along with postdiagnostic support. Adult mental health services include perinatal CBT, family interventions, and mental health assessments. However, this study will only focus on CYP services.

## Methods

As this is a small case series study that uses fully anonymized clinical record data, research ethics committee approval was not required.

### Participants

**Figure 1:**
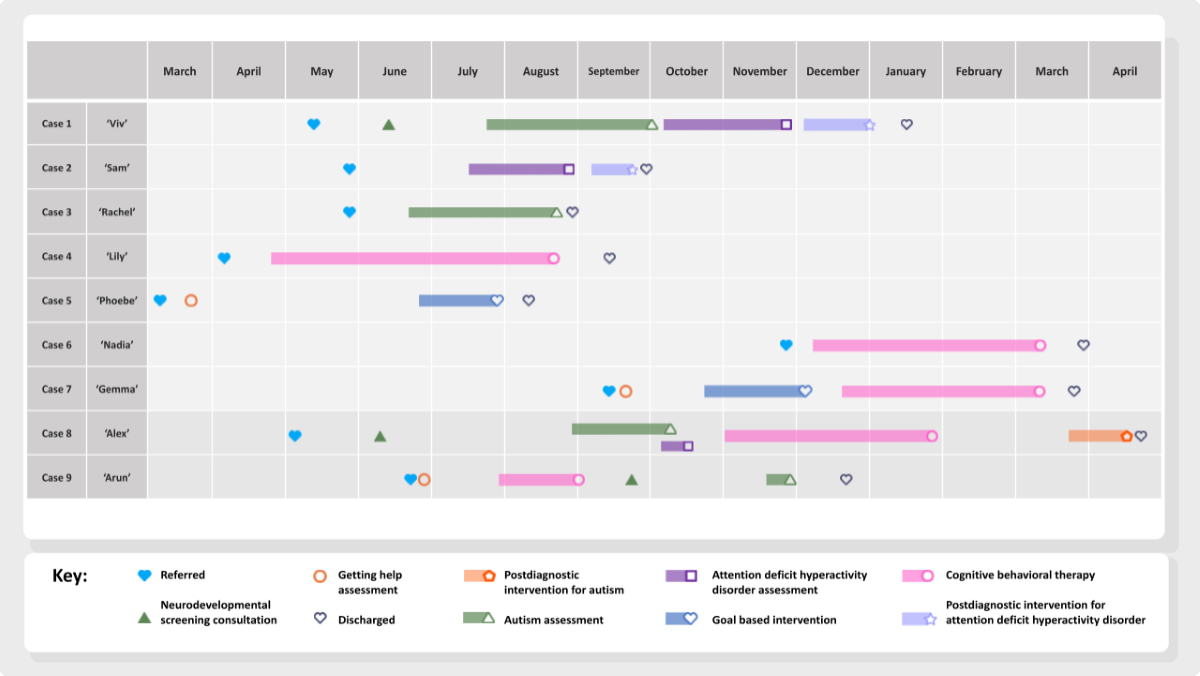
Timeline of all 9 cases through Healios’ services.

This study included 9 cases seen at Healios between April 2018 and May 2020. Of these, 6 were female and 3 were male, and they were aged 8-15 years (mean 11.67, SD 2.83; Table 1). Although this represents a higher proportion of females than referrals from 2018 to 2020 (female: 5763/10,763, 53.54%; male: 5000/10,763, 46.46%), gender balance was not expected to have any bearing on the results, as cases were selected to be representative of different service lines available to CYP at Healios. Further details on case selection can be seen in the section *Case Selection and Procedure*. The journeys of the 9 cases through Healios’ services are shown in Figure 1.

**Table 1:** Participant overview.

Case number	Pseudonym	Sex	Age (years)	Presenting concerns in order of presentation or identification	Number of sessions	Number of services	Length of time from referral to first appointment (days)	Length of time with service from first appointment to discharge (weeks)^a^
1	Viv	Male	12	Autism and ADHD^b^	10	4	30	32
2	Sam	Male	9	ADHD	7	2	51	11
3	Rachel	Female	11	Autism	4	1	24	9
4	Lily	Female	14	Low mood and self-harm	13	1	22	20
5	Phoebe	Female	15	Low mood, low self-esteem, and minor self-harm	7	2	11	20
6	Nadia	Female	13	Anxiety and panic attacks	10	1	9	17
7	Gemma	Female	15	Low mood, low self-esteem, self-harm behaviors, and anxiety	17	3	4	27
8	Alex	Female	8	Autism, ADHD, and anxiety	20	5	35	46
9	Arun	Male	8	Anger, aggression, and autism	8	4	18	26

^a^Rounded to nearest week.

^b^ADHD: attention-deficit/hyperactivity disorder.

### Case Selection and Procedure

We used a multiple case–study design with 9 cases identified using convenience sampling as representative of the service lines offered by Healios [19]. To examine the different pathways that clients could take through Healios, researchers identified contracts where multiple service lines had been commissioned, and team leads were approached to think of relevant cases. Inclusion criteria were patients who were aged <16 years, had completed their full course of treatment or assessment, and whose case had been closed by Healios (ie, had completed feedback questionnaires and, where appropriate, had completed routine outcome measures [ROMS]). Cases were randomly selected from relevant NHS contracts, and case notes were checked against the inclusion criteria. Once cases that covered a range of the services available to CYP at Healios as well as the different pathways that could be taken were identified, no further cases were sought. The relevant outcomes and process data were then extracted. Key themes and elements of web-based work were identified from Stoll et al [10] and Nicholas et al [9] to provide structure for the data extraction during which new themes were added and existing themes reanalyzed and recategorized. Following this iterative process, 10 factors were identified (Textbox 1).

**The 10 key elements identified for data extraction.**

**Key elements (KEs) and evidence**

- KE1: Flexible delivery and timely response
  Availability of weekend or evening appointments; provision of short waiting times
- KE2: Personalized care to the individual
  Provision of person-centered care; tailored content; referrals to different pathways
- KE3: Comprehensive care enabled by multiple interconnected services
  Provision of referrals between different pathways within Healios
- KE4: Effective client engagement and productive therapeutic alliances
  Analysis of Healios post session–ratings data
- KE5: Use of multiple communication tools
  Use of videoconferencing; provision of therapeutic information, interactive whiteboards, in-session rating scales and outcome measures
- KE6: Client satisfaction with the service
  Analysis of the Friends & Family Test and the Healios Experience of Service Questionnaire data
- KE7: Good clinical outcomes
  Analysis of routine outcome measures and goals
- KE8: Ease of family involvement throughout sessions or from different locations
  The young person being joined in sessions by family members, or questionnaires completed by the family and the young person
- KE9: Facilitation of multi-agency working and integration with National Health Service (NHS) services
  Integration into local NHS pathways, automated reporting, school input into assessments
- KE10: Management of risk and safeguarding
  Addressing of the risk or safeguarding concerns on the web

## Results

The 10 key elements (KEs) identified for the web-based service provision are presented below. These have been identified through previous literature [9,10], with novel themes added and existing themes recategorized, as appropriate during data extraction.

### KE1: Flexible Delivery and Timely Response

Healios offers services between 9 AM and 8 PM, Monday-Sunday, giving young people and their families maximum flexibility in scheduling sessions via their web-based portal. Waiting time from referral to initial appointment ranged from 4 to 51 days (mean 22.67, SD 14.59). For comparison, in-house NHS autism assessments have an average waiting time of 352 days from referral to diagnosis [20], and some local CAMHS mental health services report a median waiting time of 82-182 days with an overall average waiting time of 56 days for treatment [21]. The total time from referral to discharge for the case-study group was between 18 and 51 weeks (mean 26.35, SD 11.56).

Gemma had her first appointment booked on the day her referral was accepted and was seen 4 days later. Similarly, Nadia was booked in for her first appointment within a day of her referral being accepted and was seen 9 days later (see Table 1 for all wait times from referral to first appointment). Lily booked the majority of her sessions for weekday evenings and stated in her first session that part of the appeal of Healios was that she could still attend school as usual with time for therapy in the evenings. Alex and Arun also scheduled evening sessions; Alex did so to coordinate the appointments with his father’s working hours.

Communication between Healios and myself was really good, appointments were made to accommodate my rota at work, my daughter felt at ease speaking with the clinicians.

Alex’s father

It was good not having the long waits that have been happening with CAMHS. Was good to be able to do online sessions so as to reduce anxiety of my daughter and really useful during COVID-19 to be able to complete sessions.

Rachel’s mother

Being able to be at home in my own surroundings where I felt at ease.

Gemma

### KE2: Personalized Care to the Individual

All session content was adapted to suit the young person, and this was evident in all 9 exemplar cases. Gemma was referred for a getting help assessment, where she disclosed severe low mood and social anxiety symptoms, and was referred on to GBI. She made progress but required further support and was stepped up to CBT. She was initially anxious about being on camera, and the clinician suggested using a post-it note to block the camera. After further sessions, she was more comfortable with the camera switched on and felt that she was able to appear on screen. Alex was initially referred to the ND team but showed symptoms of anxiety and reported intrusive thoughts during her ND screening and autism assessment; she was referred to the Mental Health CBT Pathway before starting her PDI sessions. Phoebe was seen for a getting help assessment and was booked for her first session of GBI in early May, but this was postponed until the end of June because of exam commitments. Viv said,

It was just amazing they understood me.

Viv

### KE3: Comprehensive Care Enabled by Multiple Interconnected Services

Healios offers multiple service lines and, where commissioned, can move referrals between these services as appropriate. Hence, young people experiencing multiple difficulties can receive comprehensive care through interconnected digital pathways, and 6 of the 9 cases received multiple services. Arun was initially referred for a getting more help assessment and subsequent CBT, but because of lack of engagement in the early sessions, he was internally referred for ND screening. Similarly, Alex was referred to Healios for an ND screening and was assessed for both autism and ADHD. However, during these sessions, she presented with symptoms of anxiety and intrusive thoughts and was also referred to within Healios for CBT sessions in addition to PDI for autism.

As part of Healios’ mental health services, getting help and getting more help assessments are offered before treatment to explore current difficulties, early life experience and development, and to assess risk. Both Phoebe and Gemma were referred to Healios for a getting help assessment, following which both were offered 6 sessions of GBI, with Gemma being further stepped up to CBT on completion of GBI.

As demonstrated by these cases, web-based delivery enables flexible and efficient provision of services while meeting individual needs with an average waiting time between services of 34 days (SD 30.39; median 31, range 13-98).

Very friendly, knowledgeable and helpful staff who listen and really care. There was very little time to wait between appointments, and we went through the process very quickly. I would recommend Healios to anyone.

Arun’s father

### KE4: Effective Client Engagement and Productive Therapeutic Alliances

Effective client engagement and therapeutic alliance was examined using Healios postsession ratings (HPSR). At the end of each session, clients rated 4 statements on a scale of 0-100.

In 6 cases, HPSR was used. With Nadia and Alex, HPSR was used for every session of CBT, and with Viv, it was used for all sessions of PDI for ADHD; however, with Gemma and Arun, it was only used sporadically. Table 2 summarizes the average scores for each statement given by the individual cases in which the HPSR was used.

For these cases, the average score given on the *overall* measure of HPSR was 92.72 (SD 12.63; range 68.53-100), indicating that sessions are appropriate, and the average subscale scores of >90 suggest productive therapeutic alliances. In particular, Viv and Alex consistently rated their sessions as 100, indicating that they formed strong therapeutic alliances with their clinicians and were engaged with their sessions and treatment. This was reflected in the feedback given by Viv’s mother, “Listen, gave constructive advice, didn’t shy away from giving tough messages.” Nadia also commented on the therapeutic alliance formed, “They listened to me, took interest in what I had to say and always had a positive response.”

Very friendly and approachable. Managed to get my son engaged.

Arun’s father

They spoke directly to my child making him feel valued.

Viv’s mother

**Table 2:** Average HPSR^a^ scores by case.

HPSR scales	Case					
	Viv^b^	Lily^c^	Nadia^d^	Gemma^e^	Alex^d^	Arun^f^
Listened to (0=“Did not listen to me”, 100=“Listened to me”)	100.00	99.03	100.00	85.64	100.00	99.90
Importance (0=“What we did and talked about was not really that important to me,” 100=“What we did and talked about were important to me”)	100.00	93.18	99.72	74.87	100.00	99.97
What we did (0=“I did not like what we did today,” 100=“I liked what we did today”)	100.00	87.47	99.62	78.13	100.00	100.00
Overall (0=“I wish we could do something different,” 100=“I hope we do the same kind of things next time”)	100.00	88.83	98.93	68.53	100.00	100.00

^a^HPSR: Healios postsession ratings.

^b^Healios postsession ratings completion rate: 100% (3/3).

^c^Healios postsession ratings completion rate: 46% (6/13).

^d^Healios postsession ratings completion rate: 100% (10/10).

^e^Healios postsession ratings completion rate: 70% (7/10).

^f^Healios postsession ratings completion rate: 75% (3/4).

### KE5: Use of Multiple Communication Tools

All sessions take place on Healios’ clinical platform and involve interactive activities, including completing formulations, therapeutic exercises, rating scales, and using a whiteboard. The use of these features was evident in all 9 cases. Phoebe used the interactive whiteboards during her getting help assessment to describe her current feelings and how she found school, friendships, and home life using emojis and pictures. Lily used the mood diary between sessions to document her thoughts and feelings, capturing when she had felt worried, which were subsequently shared with her clinician in the next session.

In addition to HPSR, the interactive platform includes sliding scale measures for in-session self-rating of mood and the severity of difficulties. The platform also allows clinicians to share other resources in real time (eg, looking at websites) and for Arun, this included playing a web-based, interactive version of *Connect4* at the beginning of his autism assessment to promote engagement.

Question 12 of the Healios Experience of Service Questionnaire (HESQ)—“The online interactive activities during my session were helpful”—provides some feedback on the interactive communication tools. Unfortunately, 2 of the 5 young people who completed the HESQ answered “Don’t know” but 2 answered “Certainly true” and 1 answered “Partly true.” Furthermore, 4 of the 5 parents who completed this question responded “Certainly true” and 1 responded “Don’t know.” We do not have data from the other cases because of a technical hitch with this questionnaire, but it is worth noting that in response to this question, for the service as a whole, 66.7% (1917/2874) of parents and young people responded “Certainly true;” 18.93% (544/2874), “Partly true;” 2.75% (79/2874), “Not true;” and 11.62% (334/2874), “Don’t know.”

The staff were nice. I liked the part where we had 1:1 sessions and enjoyed the activities that we did.

Rachel

### KE6: Client Satisfaction With the Service

All cases reported satisfaction with the services received. Feedback was routinely collected from young people and their families at the end of assessment or treatment using the NHS Friends and Family Test (FFT) and the HESQ. The FFT asked if they would recommend the service to others, with responses recorded on a 5-point scale (young person version: 0=“I disagree a lot” and 4=“I agree a lot”; parent version: 0=“Extremely unlikely” and 4=“Extremely likely”). The HESQ comprised 12 items, 9 relating to satisfaction with the care received and 3 to the web-based processes or environment, all being rated on a 3-point scale (0=“Not true,” 1=“Partly true,” and 2=“Certainly true”). Both questionnaires also invited comments on things that went well and suggestions for improvement. In all cases, either the FFT or HESQ had been completed by the young person and/or their parents, and in some cases completed multiple times after different services. In 6 cases, both the FFT and HESQ were completed (Table 3).

Sam answered 8 HESQ questions on care and 2 on environment, answering “Certainly true” for all questions except “It was easy to talk to the people who saw me and If a friend needed this sort of help, do you think they should come here?” to which he answered, “Partly true.” Both Sam and his mother responded to the same 10 of the 12 questions, not answering “Were you given enough explanation about the help available here?” and “The online activities in my sessions were helpful.” Sam’s mother responded “Certainly true” to all the other questions. It is not clear why they did not answer 2 of the HESQ questions, but it is interesting to note that Sam’s mother also completed the FFT and responded that it would be “Extremely likely” that she will recommend the service to others. She also wrote an unsolicited letter in which she thanked the clinician for her *“*professionalism and kind heart,” saying that it was “like having a genie in a bottle: she recommended so many improvements as if she’d known my son all his life.”

Gemma answered 4 HESQ care questions, rating “I have been given enough explanation about the help available here” as “Certainly true” and “I feel that the people who saw me listened to me, My views and worries were taken seriously,” and “Overall, the help I have received here is good” as “Partly true.” It is not clear why she did not answer the other questions, but in the free text she added that “Being understood, non-judgemental” was what she liked about the care. Gemma did not complete the HESQ following her CBT and GBI, but her mother completed the HESQ for all services and responded “Certainly true” to all questions.

Engaging Healios was one of the best decisions I’ve made in my life...Because of Healios, there is now light at the end of the tunnel.

Sam’s mother

**Table 3:** FFT^a^ and HESQ^b^ responses by case.

Case	Service feedback given for	FFT		HESQ (maximum score of 24 if all 12 questions are completed)	
		Young person	Parent	Young person	Parent
Viv	ND^c^ screen	“I agree a lot”	“Extremely likely”	—^d^	—
Viv	PDI^e^ for ADHD^f^	“I agree a lot”	“Extremely likely”	23/24 (17/18 + 6/6)	24 (18/18 + 6/6)
Sam	ADHD assessment	—	“Extremely likely”	18/20 (14/16 + 4/4)	20/20 (16/16 + 4/4)
Rachel	Autism assessment	“I agree a lot”	“Extremely likely”	—	—
Lily	CBT^g^	“I agree a lot”	—	22/22 (18/18 + 4/4)	—
Phoebe	Getting help assessment	—	“Extremely likely”	—	—
Nadia	CBT	“I agree a lot”	“Extremely likely”	24/24 (18/18 + 6/6)	24/24 (18/18 + 6/6)
Gemma	Getting help assessment	“I agree a lot”	“Likely”	10/14 (5/8 + 5/6)	24/24 (18/18 + 6/6)
Gemma	GBI^h^	—	“Extremely likely”	—	24/24 (18/18 + 6/6)
Gemma	CBT	—	“Extremely likely”	—	24/24 (18/18 + 6/6)
Alex	ND screen	—	“Extremely likely”	—	—
Arun	Getting more help assessment	—	“Extremely likely”	—	22/22 (16/16 + 6/6)
Arun	ND screen	—	“Extremely likely”	—	—
Arun	Autism assessment	—	“Extremely likely”	—	23/24 (18/18 + 5/6)

^a^FFT: Friends and Family Test.

^b^HESQ: Healios Experience of Service Questionnaire.

^c^ND: neurodevelopmental.

^d^The questionnaire had not been completed.

^e^PDI: postdiagnostic intervention.

^f^ADHD: attention-deficit/hyperactivity disorder.

^g^CBT: cognitive behavioral therapy.

^h^GBI: goal-based intervention.

### KE7: Good Clinical Outcomes

Standardized assessment measures were used as part of clinical and diagnostic assessments and to measure therapeutic progress. The Revised Children’s Anxiety and Depression Scale (RCADS) and Strengths and Difficulties Questionnaire (SDQ) was used with all the cases, except with Sam, where only the SDQ was completed because of technical problems. In GBI and CBT cases, the RCADS [22,23] and SDQ [24] were completed before and after the course of intervention as outcome measures, with any missing data being related to parental noninvolvement after initial assessment in the case of older children. There is also clear evidence that session-by-session outcome measures, Young Person Clinical Outcomes in Routine Evaluation (YP-CORE) [25], and goal-based outcomes (GBOs) [26] were successfully used to complement the RCADS and SDQ, where there was a risk of incomplete data. GBOs assessed progress toward idiosyncratic therapeutic goals using a 0-10 scale, whereas the YP-CORE, a 10-item self-report questionnaire, covered anxiety, depression, trauma, physical problems, functioning, and risk to self. This is scored on a 5-point scale (0=“not at all” and 4=“most or all of the time”). YP-CORE total scores up to 5 are considered healthy; 6-10, low level; 11-15, mild; 16-20, moderate; 21-25, moderately severe; and above 25, severe problems (Table 4). In most cases, the outcome measures detected reliable and/or clinical improvement (reliable improvement was considered as statistically significant change and clinical improvement as scores changing from above to below the clinical cut-off).

**Table 4:** Available outcome data by case.

Questionnaire and timepoint		Case					
		Lily	Phoebe	Nadia	Gemma		Alex
**RCADS^a^ self**							
	Before treatment	73 (clinical)	61 (normal)	69 (borderline clinical)	78 (clinical)		62 (normal)
	End of treatment	54 (normal)^b,c^	53 (normal)	33 (normal)^b,c^	61 (normal)^b,c^		54 (normal)
**RCADS parent**							
	Before treatment	65 (borderline clinical)	67 (borderline clinical)	77 (clinical)	—^d^		87 (clinical)
	End of treatment	—	—	51 (normal)^b,c^	79 (clinical)		79 (clinical)
**SDQ^e^ self**							
	Before treatment	20 (very high)	21 (very high)	22 (very high)	23 (very high)		—
	End of treatment	9 (average)^b, c^	—	8 (average)^b,c^	17 (raised)^b^		—
**SDQ parent**							
	Before treatment	12 (average)	13 (average)	22 (very high)	—		24 (very high)
	End of treatment	—	—	13 (average)^b,c^	18 (high)		26 (very high)
**Goals**							
	When set-end of treatment						
		Goal 1: 6-9^c^	Goal 1: 3-8^c^	Goal 1: 0-10^c^	GBI^f^—goal 1: 0-2	CBT^g^—goal 1: 2-3	Goal 1: 1-6^c^
		Goal 2: 7-8	Goal 2: 2-7^c^	Goal 2: 4-10^c^	Goal 2:0-1	Goal 2:3-4	—
		—	—	Goal 3: 0-3^c^	Goal 3: 4-10^c^	Goal 3: 4-2	—
		—	—	—	Goal 4: 0-2	Goal 4: 5-3	—
		—	—	—	Goal 5:1-1	Goal 5: 0-0	—
		—	—	—	Goal 6: 0-0	—	—
		—	—	—	Goal 7: 0-1	—	—
		—	—	—	Goal 8: 0-0	—	—
**YP-CORE^h^**							
	Before treatment	—	18 (moderate)	11 (mild)	36 (severe)		18 (moderate)
	End of treatment	—	8 (low-level problems)^b,c^	0 (healthy)^b, c^	19 (moderate)^b, c^		20 (moderate)

^a^RCADS: Revised Children’s Anxiety and Depression Scale.

^b^Clinical improvement.

^c^Reliable improvement.

^d^The questionnaire had not been completed.

^e^SDQ: Strengths and Difficulties Questionnaire.

^f^GBI: goal-based intervention.

^g^CBT: cognitive behavioral therapy.

^h^YP-CORE: Young Person Clinical Outcomes in Routine Evaluation.

Gemma was initially referred for a getting help assessment, because she struggled with significantly low mood, anxiety and insomnia related to long standing social relationship difficulties and bullying at school. Her initial score of 36 on the YP-CORE indicated severe problems, but following the available six sessions of GBI, her scores had only slightly improved, and she was stepped up to CBT. When she started her CBT treatment a fortnight later, Gemma completed the RCADS and SDQ, both of which indicated clinically significant problems. Following 10 sessions of CBT that focused on understanding and coping with anxiety, especially in group situations at school, all of her outcome measures showed clinically significant improvement: her YP-CORE scores had reduced to the moderate level, the RCADS changed from *clinical* and fell within the normal range and SDQ scores reduced from *very high* to *raised*.

Alex was initially referred to the ND pathway for a screening consultation and was diagnosed with autism. During the assessment, she disclosed intrusive thoughts and feelings of anxiety, which was reflected in the anxiety subscale of the RCADS. She was referred for 10 sessions of CBT that focused on psychoeducation around anxiety and exposure to situations as well as sleep hygiene and thought challenging. At the end of treatment, Alex’s scores on the RCADS had reduced overall and the anxiety subscale score fell within the normal range, showing clinical improvement on this measure. Although Alex’s father’s score on the RCADS had reduced, they still fell within the clinical range. No improvement was seen on the SDQ, which was also completed by him. YP-CORE scores also remained consistent, suggesting that Alex was still experiencing moderate problems. This may be related to her autism and, therefore, was addressed in her subsequent PDI.

Idiographic measures such as GBOs are highly sensitive to therapeutic change [27], and an improvement of 2.45 points represents a reliable change [28]. Phoebe set 2 goals in GBI session 1 and made steady progress throughout the 6 sessions. Her first goal, “Be more confident,” increased from a score of 3 to 8, and her second goal, “To self-harm less,” increased from a score of 2 to 7, indicating reliable change on both by session 5 (given that the primary focus was on relapse prevention, goals were not scored in the final session). Phoebe’s improvement on GBOs was mirrored by her steadily decreasing scores on the YP-CORE from 18 (moderate problems) to 8 (low-level problems). In contrast, Lily set 2 goals in her fifth session of CBT, and her goal ratings fluctuated during treatment. Her first goal was to challenge her thoughts, and although this was initially rated as 6, it decreased to 4 in her eighth session, during which she reported that she had experienced exam-related stress. During her final 13th session, she rated this goal as 9. Her second goal, “to be less self-critical,” was initially rated 7 and remained steady before being rated 6 at week 12 and then 8 in her final session. Therefore, Lily exhibited reliable change on her first goal only while her RCADS and SDQ scores showed significant clinical improvement, that is being within the normal range at the end of treatment.

Nadia was also referred to Healios for CBT and set goals in session 3. Her first goal was to be more proactive after school and on weekends. This goal was scored 0 when set but it increased throughout the course of CBT and was scored 10 (fully achieved) in her final (10th) session. Her second goal was to get herself ready for mathematics lessons; this goal, which was initially scored 4, was fully achieved by session 5. Nadia also aimed to have multiple horse-riding lessons and set this as her third goal; this goal was scored a 0 when set and increased to 3 in her final session. Therefore, Nadia showed reliable change on all her goals. Despite Nadia’s significant school-related difficulties detected in the first session, her YP-CORE score was 11, putting her in the mild problems range. Her RCADS and SDQ scores indicated clinically significant problems, which were mirrored by her mother’s scores (Table 4). Although her YP-CORE scores fluctuated between 2 (healthy) and 8 (low-level problems) week after week between sessions 2 and 9, they dropped to 1 in her fourth session. This session took place over the Christmas holidays and Nadia reported reduced anxiety because she did not have to attend school. Despite her YP-CORE scores increasing again after the Christmas period, by the end of treatment, she had returned to school and her YP-CORE score was 0; her RCADS and SDQ scores were in the normal range.

Already, ‘Sam’ finds it easier to fall asleep and his self esteem has improved.

Sam’s mother

It is such a relief to finally have some answers as to why ‘Arun’ has been struggling so much.

Arun’s father

### KE8: Ease of Family Involvement Throughout Sessions or From Different Locations

In all 9 cases, there was evidence that parents were involved in some or all of the sessions. Some of the older children had their parent or parents attending either the beginning of the sessions or just their first session. There was also evidence in all cases of parents completing ROMS and feedback questionnaires.

Both cases 4 and 6 had parental involvement throughout their CBT, with Nadia’s mother joining her throughout the 10 sessions of CBT. In contrast, Lily was joined by her mother for only 3 of her 13 CBT sessions. This demonstrates the collaborative nature of the sessions, with Lily being able to choose when her mother could join her sessions. In both cases, however, family was found to be a protective factor. Both the young people reported that when they were struggling between sessions, they felt that they were able to speak to their mothers for support.

In Arun’s case, his father’s involvement appeared integral, and he attended all the sessions. Arun was initially referred for CBT, following a getting more help assessment; however, after the first few sessions, it became apparent that Arun was not engaging with treatment. Despite this, Arun’s father attended the sessions and spoke about the impact that Arun’s current difficulties were having on the whole family. After talking this through with their Healios CBT therapist, it was decided to refer Arun to the ND pathway for assessment. Had it not been for Arun’s father’s involvement, it may have been more challenging to understand his difficulties and what services he required and following his disengagement from CBT, he may have been discharged without further assessment.

When Gemma’s mother joined for her first session of GBI, Gemma felt comfortable to attend the subsequent sessions on her own. For sessions 2-6, her mother only joined for the first few minutes to give the clinician an update on their week before leaving Gemma and the clinician to work together.

Whereas the waiting lists for CAMHS were very lengthy, our experience with Healios has taken but a couple of months. For that, we thank you because much more waiting could have resulted in the break up of our family as we were at crisis point.

Arun’s father

### KE9: Facilitation of Multi-Agency Working and Integration with NHS Services

Web-based working facilitates multi-agency working and the ability to integrate with NHS services. There was evidence of integration with NHS services in all 9 cases of this study. In all the cases, detailed reports were written on completion of the service and shared directly with the referring CAMHS services and general practitioners.

For autism and ADHD services, clinicians benefit from the digital clinical records platform, which pulls the assessment scores into a template report. This means that clinicians can input assessment scores once only; this prevents errors in the writing of the bespoke report. Once completed, the report is automatically shared with the referring services through their referral portal and the family through their own portal. This process was followed in cases 1, 2, 3, 8, and 9.

In cases where multiple services had been received, integration with the NHS was vital to ensure that local teams were kept up to date with the progress of the young person. For example, in Arun’s case, detailed reports were written up following his getting more help assessment, CBT, ND screening consultation, and autism assessment. These contained an overview of what had been discussed and the agreed upon next steps. All 4 of these reports were shared with the referring CAMHS services, so they were aware and up to date on Arun’s care. Arun and his parents could also access these reports through their portal.

Integration with the NHS is also important to ensure that care continues seamlessly with local teams where necessary. This was important in Gemma’s care, as after receiving both GBI and CBT, her clinician thought that it might be helpful for her to be offered further intervention to build on the progress that she had made. The local CAMHS team was updated on her treatment through a comprehensive summary report and they agreed that a medical review appointment would be useful to inform the next steps and ensure that Gemma continued to receive the support she needed.

School input is sought as part of the ND services provided by Healios. This input from teachers and special educational needs coordinators offers crucial insight into young people’s behavior while at school. This was particularly important in Alex’s ADHD assessment. Although her parents reported her to be very hyperactive at home with difficulties sleeping, her teachers described her as more withdrawn and shy at school. One of her teachers also completed the Conners-3 questionnaire, giving her *average* scores on all subscale measures of inattention, hyperactivity or impulsivity, learning problems, defiance or aggression, and relationships. Consequently, the patient did not meet the criteria for a diagnosis of ADHD.

As part of Viv’s autism assessment, his school was sent a questionnaire to provide information on their current concerns and insights into his peer relationships, behavior, communication, and academic attainment. As his assessment occurred during the school summer holidays, the school did not submit their feedback until the term started again. This meant that there was a delay in his autism assessment outcomes. Once this feedback was received, the multidisciplinary team was able to meet to discuss all the information and arrive at a conclusion. The school then also provided input for Viv’s ADHD assessment and completed the Conners-3 questionnaire. Both of these assessment reports were written, including the school’s input, and shared with the referring CAMHS service as well as with Viv and his family. Families are encouraged to share these reports with the school to ensure that they can support the young people.

### KE10: Management of Risk and Safeguarding

A commonly expressed concern regarding web-based assessments and treatment is that clinicians might not be able to accurately assess and effectively manage risk and safeguarding concerns. Healios follows strict risk and safeguarding protocols to manage any risks arising during sessions and communicates closely with the referring NHS and safeguarding teams when a local response is required.

All 9 cases underwent a risk assessment, but only Lily presented with concerns that required further action. She disclosed previous self-harm and suicidal thoughts and a current plan to end her life but no intent to do so. Due to the historical nature of suicidal ideation, no action was taken at this stage, but the risk was monitored in each session. In the middle of Lily’s treatment, her mood dropped and her mother joined a session to disclose worries of risk to self over the previous week. Lily said that she had been having thoughts of being worthless and suicidal ideation; however, she reported no intent to act on these thoughts. During this session, a crisis management plan was formed, and protective factors and distraction techniques were discussed for Lily to use when feeling low. The Healios clinician was able to share the number for the local CAMHS crisis team if Lily’s mother had any concerns about Lily’s well-being between sessions. A risk assessment form was completed, the Head of Mental Health Services at Healios was informed, and the risk assessment and management plan was shared with the local CAMHS service. Risk was then monitored closely for the remaining 8 sessions.

In cases 1, 5, 7, and 9, lower-level concerns around risk were identified during risk screening and crisis phone numbers were shared for families to use if they had any concerns between sessions. In addition, a risk capture form was completed and attached to the session summary so that it could be accessed by the referring CAMHS team.

## Discussion

### Principal Findings

The purpose of a RE is to assess whether a program or service works in a particular context. In this study, we analyzed 9 cases that had received a digital mental health or ND service via the Healios web-based platform to verify our *mid-range theory* and our CMO statements.

We identified 10 KEs:

- KE1: Flexible delivery and timely response;
- KE2: Personalized care to the individual;
- KE3: Comprehensive care enabled by multiple interconnected services;
- KE4: Effective client engagement and productive therapeutic alliances;
- KE5: Use of multiple communication tools;
- KE6: Client satisfaction with the service;
- KE7: Good clinical outcomes;
- KE8: Ease of family involvement throughout sessions or from different locations;
- KE9: Facilitation of multi-agency working and integration with NHS services;
- KE10: Management of risk and safeguarding.

These elements supported the 6 CMOs: There was clear evidence that young people and their families valued the responsiveness and flexibility of the web-based mental health and ND services and, in particular, how quickly they were seen. There was also clear evidence of individual needs being met, good therapeutic alliances, and client satisfaction. Multiple communication tools appeared to maximize engagement and working on the internet facilitated multi-agency communication and delivery of safe care. The above factors may be related to the finding of good clinical outcomes, but the methodology of this study does not allow any conclusions to be drawn regarding causality. Nonetheless, this study has verified our program theory regarding digital mental health and ND services and provides useful insights for the further development of digital services.

### Limitations

As this is qualitative research using a convenience sample, albeit cases selected in a random fashion to provide data in line with the aims of the study, these results are not necessarily representative of the service as a whole, and the results cannot be generalized to other digital services. For example, this study does not explore nonengagement or dropout from digital services [29-31], and other digital providers using other technical platforms and procedures may not realize equivalent advantages. In addition, all cases explored were CYP; therefore, the mechanisms detailed here may not be applicable to adult populations. Studies of this type are potentially open to selection bias, as we only report 9 cases. However, this methodology was appropriate to identify the KE of web-based service delivery and explore whether interconnected digital mental health and ND services can be effectively delivered.

### Potential Advantages of Web-Based Services

The 9 cases demonstrate the potential advantages of web-based service delivery for the collection of outcome and satisfaction data. Although there were some missing data, which appeared to be the result of a range of factors including system failures, the data available were more comprehensive than that available in most in-person services. It is suggested that information technology infrastructures would help with this problem [32,33]. Collecting ROMS is quick and easy to perform on the web. Clients can complete measures in privacy in the *electronic waiting room* before the sessions, and interactive measures can also be used during sessions. Digital delivery maximizes the potential for meaningful integration of outcomes in the clinical process and the delivery of feedback-informed treatment [34].

These cases demonstrate that the web-based delivery of services also enables the appropriate involvement of family members or significant others, with people being able to attend from different locations. In all 9 cases, parents attended one or more sessions as decided by the young person, which enhanced their sense of autonomy. Parents appreciated the way in which they were able to join sessions, often to suit their work commitments. There was also evidence of the sessions being scheduled to fit around the young person’s school commitments, although some other young people had chosen to have their sessions while at school (with their school’s support). Although none of these cases involved peers in the sessions, this would be another potential advantage of web-based services.

It appears that web-based services have the potential to engage young people who might not otherwise be able to access in-person services and that the provision of having web-based services has many advantages. Another advantage that these particular cases have clearly demonstrated is the ability to offer interconnected digital services and to integrate services with the NHS. Working digitally enables seamless coordination and efficient delivery of care.

### Comparison With Prior Work

Although the effectiveness and acceptance of digital mental health solutions have been widely researched [7,10,11], this has mainly been with adults, and there have been calls for further research into this method of delivery with CYP [8]. There have been no reports of digitally native mental health and ND services.

Initial data extraction was informed by previous studies [9,10] identifying various elements of digital service delivery. Our study confirmed these elements within Healios’ services and identified additional key themes, such as multi-agency working and integration with the NHS. This study also highlighted the advantages of interconnected digital services and digital delivery of ROMS.

### Conclusions

This study demonstrates the effectiveness of interconnected digital mental health and ND services, and how web-based delivery enables young people and their families to access assessments and interventions in a more timely, flexible, and person-centered manner than in-person delivery. These 9 cases from the established digital mental health and ND services provided by Healios illustrated the potential afforded by web-based delivery beyond COVID-19 restrictions. We hope that the 10 elements identified will inform future research and facilitate the delivery of high-quality digital mental health care and ND services.

## Abbreviations

ADHD: attention-deficit/hyperactivity disorder
CAMHS: children and adolescent mental health services
CBT: cognitive behavioral therapy
CMO: context-mechanism-outcome
CYP: children and young people
FFT: Friends and Family Test
GBI: goal-based intervention
GBO: goal-based outcome
HESQ: Healios Experience of Service Questionnaire
HPSR: Healios postsession ratings
ISO: International Organization for Standardization
KE: key element
ND: neurodevelopmental
NHS: National Health Service
PDI: postdiagnostic intervention
RCADS: Revised Children’s Anxiety and Depression Scale
RE: realist evaluation
ROMS: routine outcome measures
SDQ: Strengths and Difficulties Questionnaire
YP-CORE: Young Person Clinical Outcomes in Routine Evaluation
